# Oral lesions in patients with severe COVID-19 in the National Institute of Respiratory Diseases in Mexico: Case series

**DOI:** 10.34172/joddd.2023.37072

**Published:** 2023-04-03

**Authors:** Carlos Alberto Carrasco Rueda, Ilan Vinitzky Brener

**Affiliations:** Oral and Maxillofacial Surgeon, Stomatology Department, Instituto Nacional de Enfermedades Respiratorias “Ismael Cosio Villegas” Mexico City, México

**Keywords:** Oral lesions, COVID-19, Severe, SARS-CoV-2

## Abstract

Oral manifestations in patients with COVID-19 have already been reported in the literature. Determining whether the oral manifestations in these cases are directly related to SARS-CoV-2 infection or not has been challenging for both clinicians and researchers, although at present it has not been possible to prove. There are several possible causes for the development of the oral lesions in patients with COVID-19, among them are, opportunistic infections, drug reactions, iatrogenic and those directly related to viral infection. The present work describes the main characteristics of 10 severe COVID-19 hospitalized patients with oral manifestations. By analyzing the characteristics of the reported patients, and what is published in the literature, we conclude that for this series of cases the manifestations are not directly related to SARS-CoV-2, however, it is a possibility that should be considered for all patients.

## Introduction

SARS-CoV-2 is a neurotropic, mucotropic, and sialotropic virus that can affect the salivary glands’ function, taste sensations, smell, and oral mucosa integrity.^1^ The oral cavity is a perfect habitat for SARS-CoV-2 invasion due to the special affinity the virus has for cells with angiotensin-converting enzyme (ACE2) receptors, such as those from the respiratory tract, oral mucosa, tongue, and salivary glands. SARS-CoV-2 can alter the oral microbiota balance, which in addition to a depressed immune system, would allow opportunistic infections.^2,3^ Similarly, the intake of certain drugs could lead to salivary glands and oral mucosa alterations, responding to various injuries, and/or oral manifestations associated with COVID-19. The main ones described are xerostomia, dysgeusia, ulcers, and/or aphthous lesions. The latter can appear as multiple superficial ulcers with erythema, erythematous halos, and yellow-white pseudo-membranes on both mucous membranes, keratinized and non-keratinized. Aphthous lesions with necrosis and hemorrhagic crusts have been described to manifest more regularly in older adults with immunosuppression and severe COVID-19 infection; one hypothesis for the development of aphthous lesions and/or ulcers is given due to the ACE2 receptor and the SARS-CoV-2 interaction, which could alter the epithelial lining of salivary glands and keratinocytes, causing lesions in the oral cavity.^4^ At the same time, different etiological factors such as infections, immune system alterations, and direct trauma to the oral mucosa or epithelium,^5^ may be related to the stress of a prolonged hospital stay.^6^ Including pressure in the oral cavity conditioned by the prone position, malposition of the endotracheal tube (mainly in the corners of the lips),^7^ medication-related nutritional deficiencies^8^ such as lopinavir, and ritonavir, oseltamivir, hydroxychloroquine, among others.^9-12^ Thrombotic vasculopathy secondary to COVID-19 has also been described, induced by system mediators in the microvascular walls, which impairs endothelial cells, and activates coagulation factors^13^ and a possible hypersensitivity reaction of the mucosa to the presence of SARS-CoV-2 in the epithelium^14,15^; there is also the hypothesis that it could be associated with an exanthem pattern induced by the inflammatory action of the SARS-CoV-2 virus,^16^ presented as increased levels of cytokines (including interleukin-1, tumor necrosis facto-α), and arachidonic acid metabolites (prostaglandins) secondary to the stem cell factor production and the basic fibroblast growth factor of keratinocytes from the basal layer, in relation to post-inflammatory pigmentations that could appear in areas previously affected by trauma or chronic inflammation.^17^

 Oral manifestations in COVID-19 patients appear, on many occasions, even before respiratory symptoms, although exanthematic lesions observed in COVID-19 patients can also be observed in other viral processes.

## Case Series

This manuscript presents the main clinical and general characteristics of 10 severe COVID-19 hospitalized patients with oral manifestations (Tables 1 and 2). Five patients will be described in detail in whom an oral biopsy was performed.

**Table 1:** The general characteristics of the patients reported in the study

**No.**	**Gender**	**Age**	**Intubation**	**Comorbidities**	**COVID- 19 Severity**	**Type of lesion**	**Location**
1	M	54	Yes	GERD, gastritis	Severe	Ulcers	Lips and corner lips
2	M	67	Yes	Smoking	Severe	Herpetiform ulcers	Corner lips and skin
3	M	48	Yes	AH, alcoholism, obesity	Severe	ulcers	Lip, tongue, gum
4	F	60	Yes	Obesity	Severe	Ulcers and swelling	Lips and corner lips
5	M	60	Yes	Smoking	Severe	Bleeding ulcers	Lip, tongue, and inner cheeks
6	M	74	Yes	AH, DM, overweight	Severe	Ulcers	Corner lip
7	M	71	Yes	AH, DM	Severe	Ulcers	Lower lip and tongue
8	M	49	Yes	Smoking, obesity	Severe	Ulcers	Corner lip
9	M	50	Yes	AH	Severe	Ulcers	Bilateral corner lip
10	M	75	Yes	DM, AH, asthma	Severe	Bleeding ulcers	Corner lip and lip

GERD, gastro esophageal reflux disease; DM, diabetes mellitus; AH, arterial hypertension

**Table 2:** The main clinical characteristics of the patients reported in the study

**No.**	**Lesion appearance time after COVID-19 diagnosis**	**Outcome** **(Deceased)**	**Histopathology**	**Drugs**
1	13 days	No	Acute inflammation	Levofloxacin, Meropenem, Enoxaparin, Acyclovir, Itraconazole Piperacillin/Tazobactam, Linezolid, Amphotericin B
2	22 days	Yes (COVID-19-related pneumonia)	Ulcerated mucosa, immunohistochemistry for cytomegalovirus	Dexamethasone, remdesivir, enoxaparin, vasopressors, meropenem, acyclovir, vancomycin
3	22 days	No	Ulcerated mucosa, chronic inflammation	Fentanyl, midazolam, propofol, furosemide, dexamethasone, norepinephrine
4	23 days	No	Acute ulcerated inflammation with granulation tissue	Dexamethasone, Linezolid, Amikacin, Enoxaparin, Ceftazidime, Acyclovir, Chloramphenicol.
5	22 days	No	Small-vessel lymphocytic vasculitis, multifocal thrombotic microangiopathy, lymphocytic vasculitis	Paracetamol, Insulin, Enoxaparin, Dexamethasone, Omeprazole, Cephalotin, Itraconazole, Midazolam Meropenem TMP/SMS
6	29 days	Yes (COVID-19-related pneumonia)		Ivermectin TSM, amphotericin piperacillin/tazobactam, omeprazole, enoxaparin, linezolid
7	32 days	Yes (COVID-19-related pneumonia)		Fentanyl, dexmedetomidine, paracetamol, furosemide, heparin, insulin, metoclopramide, acetylcysteine
8	8 days	No		Olanzapine, paracetamol, metamizole, insulin, losartan, atorvastatin, fentanyl, furosemide, voriconazole
9	28 days	Yes (COVID-19 related pneumonia)		Fentanyl, midazolam, propofol, norepinephrine, paracetamol, enoxaparania, insulin, metoclopramide, voriconazole, dexamethasone
10	26 days	No		Dexmedetomidine, paracetamol, insulin, enoxaparin, furosemide, piperacillin/tazobactam

###  Case 1 

**Figure 1:**
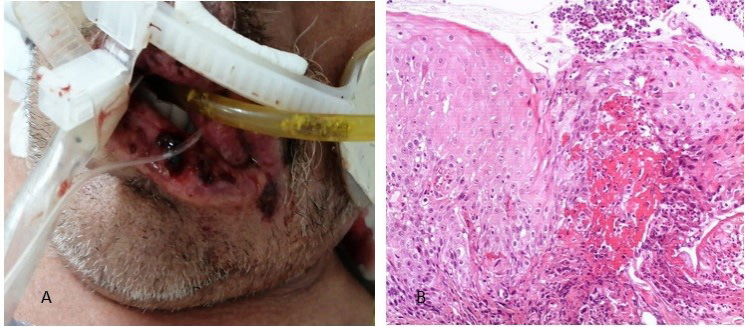
(A) Clinical appearance of the lesions. (B) Histological section stained with Hematoxylin-Eosin 10X, where the non-keratinized squamous epithelium is observed, with an ulcerated portion, detachment of the superficial layer, and some cells with cytomegaly and large nuclei, chronic inflammation, and granulation tissue.

A 54-year-old male patient with a history of severe kidney failure was admitted to the institute with signs of respiratory failure on March 24, 2021. The onset was 9 days earlier with fever, chills, and dry cough that evolved to dyspnea on small exertions, so he went to the institute where a polymerase chain reaction (PCR) test was performed, turning out positive for SARS-CoV-2, presenting low oximetry despite support with supplemental oxygen, so advanced airway management with orotracheal intubation is started, and he is hospitalized. On April 4, 2021, 10 days after admission, he begins with ulcers on the lower lip and bilateral lip corner. In the oral cavity, aphthous-type ulcers of different sizes and irregular margins are observed on the lower lip and bilateral lip corners, some with a hematic crust. A lip ulcer biopsy was taken under local anesthesia with a histopathological report of acute ulcerated inflammation and type I-II viral inclusions (Figure 1). Systemic management was started with acyclovir and topical GELCLAIRE® Oral Gel (glycyrrhetinic acid and polyvinylpyrrolidone). The lesions completely regressed after 7 days. During the hospital stay, the patient was managed with orotracheal intubation for 18 days and subsequently underwent a tracheostomy. During his stay, the patient was managed with levofloxacin, meropenem, enoxaparin, acyclovir, itraconazole, piperacillin/tazobactam, linezolid, and amphotericin B. The patient was discharged and continued his outpatient management.

###  Case 2

**Figure 2:**
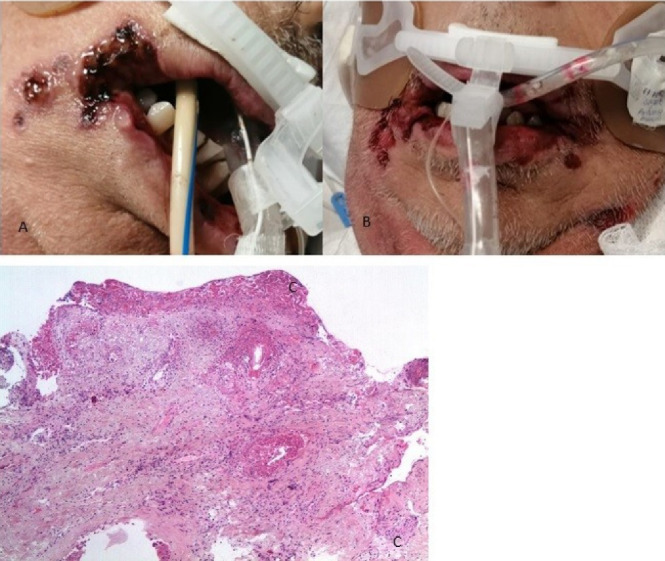
(A and B) Clinical appearance of the lesions and (C) Histological section stained with Hematoxylin-Eosin 10X, where ulcerated fibro connective tissue with the mild inflammatory process and granulation tissue is observed

A 67-year-old male patient with a positive history of smoking but no other comorbidities, who began on March 14, 2021, with symptoms of fever and dry cough that evolved to hypoxemia; went to the institute where he was evaluated confirming the COVID-19 diagnosis. Two days after admission, he presented hypoxemia despite high-flow oxygen support, so orotracheal intubation was decided to be performed. Twenty-two days after admission, the patient presented ulcers in the bilateral lip corner that extended to the lower lip and adjacent skin. We decided to perform an incisional biopsy with a histopathological result of ulcerated mucosa with a chronic inflammatory process (Figure 2), and positive for cytomegalovirus in immunohistochemical tests. Management was initiated with systemic and topical ganciclovir with GELCLAIRE Oral Gel (glycyrrhetinic acid and polyvinylpyrrolidone). During the hospital stay, the patient was managed with dexamethasone, remdesivir, enoxaparin, meropenem, and vancomycin. The oral lesions began their remission phase, however, the patient died of COVID-19-associated pneumonia and septic shock.

###  Case 3 

 A 48-year-old male patient with a relevant history of uncontrolled systemic arterial hypertension, alcoholism, and obesity. On May 24, 2021, he started with a sore throat and fever, so a quick COVID-19 test was performed giving a positive result. On June 7, he started with dyspnea and low oxygen saturation, hence the use of supplemental oxygen was indicated.

**Figure 3:**
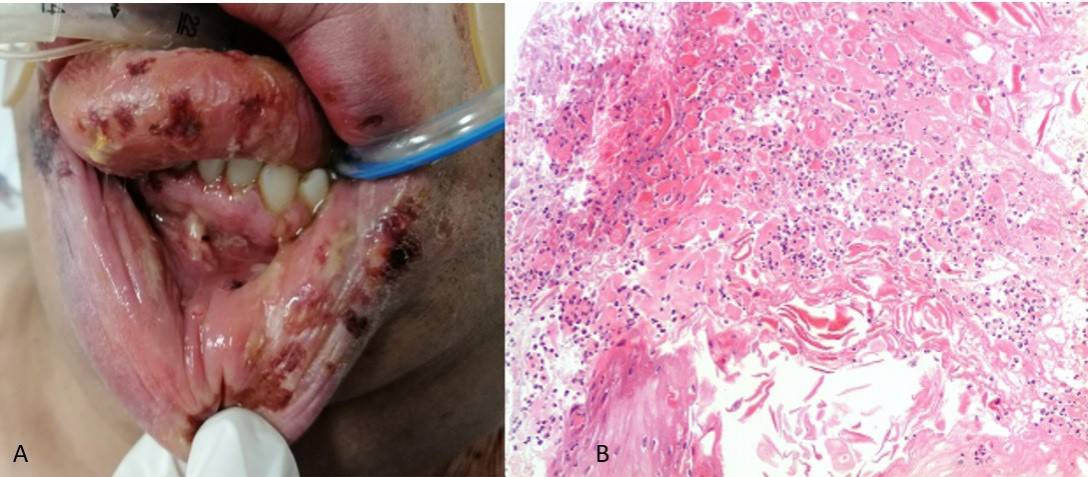
(A) Clinical appearance of the lesions and (B) Histological section stained with Hematoxylin-Eosin 10X, showing areas of necrosis, abundant PMN leukocytes, and squamous epithelial cells

On June 9, noticing a marked reduction in oximetry, he arrived at the institute where his admission for advanced airway management was decided. On June 15, 2021, during the hospital stay, he began with ulcers on his lower lip. Physical examination revealed a patient in a supine position with orotracheal intubation and orogastric tube, with aphthous-type ulcers, some of them had blood crusts of different sizes on the lower lip (both skin and mucosa), dorsum, and lateral edge of the tongue, gum, and vestibular fornix (Figure 3). Topical management with GELCLAIRE® Oral Gel (glycyrrhetinic acid and polyvinylpyrrolidone) was started and the lesions completely remitted within 7 days. During his hospital stay, the patient was managed with fentanyl, midazolam, propofol, furosemide, dexamethasone, and norepinephrine. The patient evolved successfully and was discharged 27 days after admission.

###  Case 4 

 A 60-year-old female patient with a relevant history of obesity began with general malaise and unquantified fever on March 20, 2021; on March 22 a COVID-19 PCR test was performed with a positive result. Nine days after diagnosis, the patient evolved unfavorably, presenting dyspnea on small efforts, so she went to the institute where she was assessed in the respiratory emergency service and admitted for advanced airway management due to low saturation.

**Figure 4:**
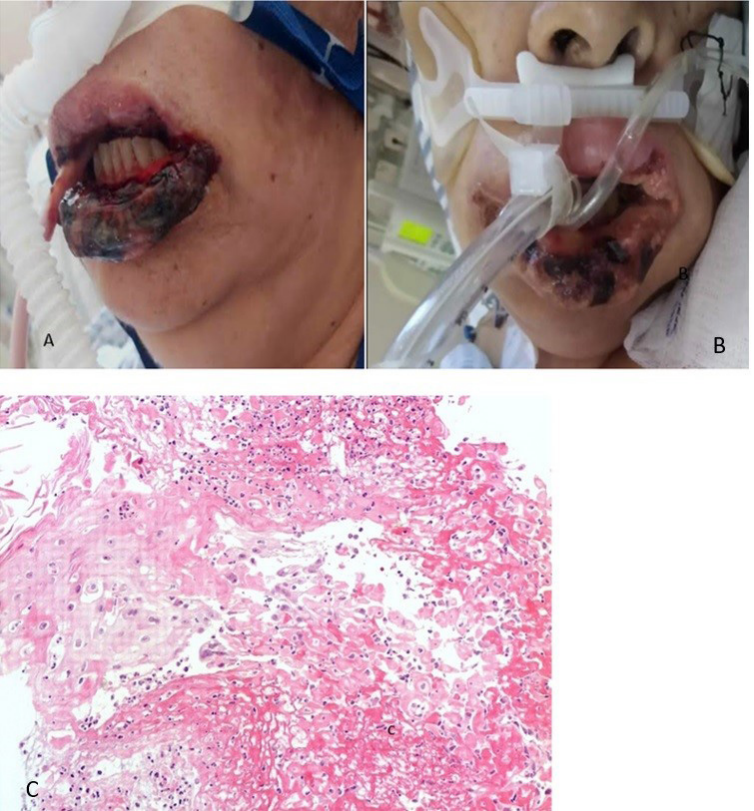
(A) Initial clinical appearance, (B) Clinical appearance after 5 days and (C) Histological section stained with Hematoxylin-Eosin 10X, showing mucosa lined by squamous epithelium with the acute inflammatory process and granulation tissue

Two weeks after her hospital admission, she began with edema in the upper and lower lips, and ulcer-like lesions appeared. Initial physical examination shows the patient in a supine position supported by high-flow nasal prongs, upper and lower lips edema and ulcer-like lesions with hematic crusts on both lips (Figure 4), topical management with steroids and GELCLAIRE® Oral Gel (glycyrrhetinic acid and polyvinylpyrrolidone) is observed.

###  Case 5

**Figure 5:**
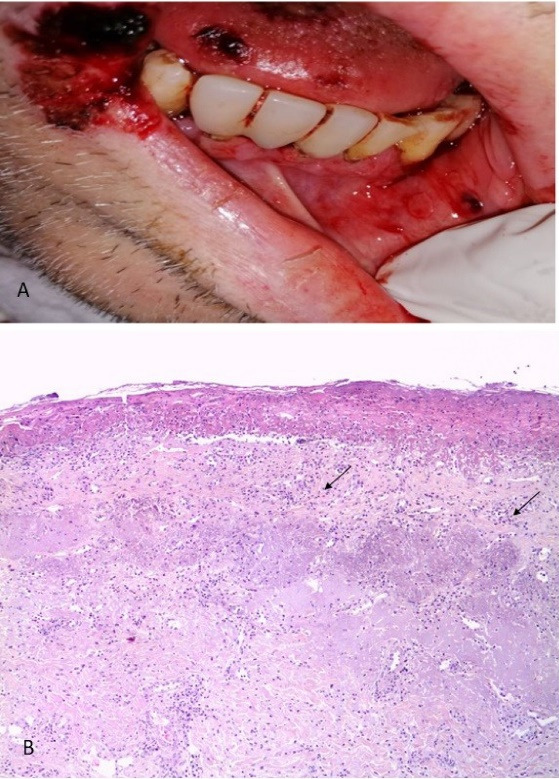
(A) Clinical appearance of the lesions and (B) Histological section stained with Hematoxylin-Eosin 10X, showing flat stratified epithelium with a fibrin-ulcerated area, acute and chronic inflammation, and formation of granulation tissue, small-vessel lymphocytic vasculitis, multifocal thrombotic microangiopathy (arrows)

A 60-year-old male patient with 30 years of smoking history, who began on April 6, 2021, with general malaise and fever, on April 7 a PCR test for SARS-CoV-2 was performed with a positive result. The patient begins with dry cough and dyspnea on small efforts, worsening as the days go by, so he goes to the National Institute of Respiratory Diseases on April 29, where he is evaluated at the respiratory emergency service, where admission for advanced airway management is decided. During his hospital stay, and 22 days after the positive COVID-19 diagnosis, the patient presented ulcers in the oral cavity, for which the dentistry department was consulted. Physical examination revealed a patient with sedoanalgesia with orotracheal intubation, and different-sized ulcers on the lower lip and the cheek mucosa, and dorsum of the tongue, some with hematic crust. Lip and tongue lesion biopsies were performed, with a histopathological result of stratified squamous epithelium with a fibrin-ulcerated area, acute and chronic inflammation, granulation tissue formation, small-vessel lymphocytic vasculitis, multifocal thrombotic microangiopathy (Figure 5). The addition of systemic acyclovir was suggested for Herpes virus-related lesions suspicion and topical management with GELCLAIRE® Oral Gel (glycyrrhetinic acid and polyvinylpyrrolidone). The lesions regressed entirely in approximately 7 days. During the hospital stay, the patient was managed with paracetamol, insulin, enoxaparin, dexamethasone, omeprazole, itraconazole, midazolam, meropenem, and trimethoprim-sulfamethoxazole.

## Discussion

The presence of dysgeusia and anosmia has been widely documented in patients with COVID-19,^18^ and a clear pathophysiological relationship for these manifestations has been established. However, the presence of lesions in the oral cavity due to the SARS-CoV-2 virus is controversial.

 This report presents the main characteristics of severe COVID-19 patients with oral manifestations. Determining whether the oral manifestations in COVID-19 patients are directly related to SARS-CoV-2 infection or not has been challenging for both clinicians and researchers, and so far a concrete answer is missing.

There are several possible causes of the development of oral lesions in COVID-19 hospitalized patients. According to Aragoneses et al,^19^ most oral lesions in COVID-19 patients are not directly related to the SARS-CoV-2 virus and can be separated into two categories:

- Opportunistic infections or co-infections involving the oral cavity, including infections such as candidiasis or herpes simplex, in the same way other debilitating diseases with a compromised immune system occur.
- Iatrogenic, including mechanical trauma-caused injuries due to prolonged intubation, and other invasive treatments (fixation methods for probes or tracheal tubes), as well as drug-induced reactions.

If these entities are discarded, it can be said that some patients will indeed present oral manifestations directly related to COVID-19.

Brandão et al,^20^ published a series of 8 cases of COVID-19 patients with oral manifestations, and described two main patterns of presentation: A) Mild pattern: young patients with mild COVID-19 in which short-lasting aphthous-like lesions occur. B) Severe pattern: older patients with moderate to severe COVID-19 with larger necrotic ulcers similar to those seen in herpes simplex type 1.

Their conclusions differ from Aragoneses et al,^19^ since based on their work they proposed oral manifestations may be directly related to the SARS-CoV-2 virus, and the incidence may be underestimated in other publications.

Amorim dos Santos et al^21^ published in 2021 a systematic review on oral manifestations in COVID-19 patients, and 6 months later an update for this review^22^ where they reported that xerostomia was the main oral manifestation, followed by dysgeusia, and subsequently oral lesions with a miscellaneous clinical aspect and a high presentation heterogeneity; since all patients (100%) in our study were intubated and sedated, xerostomia and dysgeusia were not assessed, hence it focused on oral lesions.

 In this report, lips were found as the main site for lesions appearance, with presence in 10 patients (100%), followed by tongue in 2 patients (20%), and gingiva and cheek mucosa in 1 patient (10%). Most of the published articles differ from these results since they report the tongue as the main site for lesions appearance, and patients with the mild and moderate presentation; while in our series all patients were hospitalized and had a severe presentation.

Regarding the pathophysiology of oral lesions in COVID-19 patients, Orilisi et al,^23^ related it to the high expression of ACE2 receptors in oral mucosa and the fact that their interaction with the SARS-CoV-2 virus can alter the function regulation of keratinocytes causing painful oral ulcers. In addition, the immune response to infection has been postulated as an activator of Langerhans cells and lymphocytes, thus causing vasculitis and thrombocytopenia, producing oral lesions related to vascular disorders; these could explain the symptomatology presented by our patient number 5, whose vasculitis and thrombotic microangiopathy were found through histopathological evaluation.

The fact that most of the published studies only describe clinical manifestations and few of them have histopathological studies^24^ is important to emphasize. In the present work 5 biopsies were conducted, from which 3 reported inflammation, one was positive for cytomegalovirus immunohistochemistry, and the fifth case had small-vessel lymphocytic vasculitis and multifocal thrombotic microangiopathy. It was decided to stop taking biopsies from the lesions due to risk of infection of the health workers, patients in critical condition, and histopathological results may not modify the treatment of lesions.

A fundamental consideration regarding oral cavity lesions is the use of medications. Table 2 details the main medications used during the hospital stay of the patients. Given a large number of medications patients are administered, drug-induced reactions such as erythema multiforme, angioedema, or toxic epidermal necrolysis should be considered.

About the latency period from the onset of the COVID-19 symptoms until the appearance of the oral lesions, the data could be relevant for the establishment of a causal link between COVID-19 and oral manifestations; a study published by Wu et al^25^ analyzed 51 cases and reported an average time for lesions appearance of 3.2 days from the COVID-19 onset, Mahmoud et al^26^ reported a series of cases and stated that most of the lesions occurred concomitantly with hyposmia/dysgeusia or up to 14 days later, and all oral mucosa lesions healed at the same time as the SARS-CoV-2 infection symptoms. These data contrast with the findings in our patients, where the average number of days for the appearance of the oral lesions is 22.5. Such difference may be due to all our cases having a severe COVID-19 presentation and being hospitalized, thus many manifestations could be related to causes like airway management, immunosuppression, and medications, rather than by SARS-CoV-2 infection itself.

 Concerning the treatment of the lesions, highly suspicious or confirmed cases of Herpes virus infection were managed with systemic acyclovir; for case number 2 confirmed with cytomegalovirus, management was with systemic ganciclovir. Topical GELCLAIRE® Oral Gel (glycyrrhetinic acid and polyvinylpyrrolidone) use was indicated for all patients. The remission period for all lesions was within 10 to 14 days.

Through analysis of the patients presented here and characteristics like severity, airway management, medications, and lesions appearance time, it is considered that manifestations in most cases are not directly related to SARS-CoV-2 infection as Aragoneses et al^19^ found in their literature review, nonetheless manifestations directly related to viral infection in COVID-19 patients can not be ruled out.

## Conclusion

 The present review of cases found no histological evidence of the SARS-CoV-2 virus presence in the performed biopsies, consequently, a direct connection between the virus and oral manifestations cannot be concluded.

 Patients with severe COVID-19 infection may develop manifestations in the oral cavity; these lesions presentation is heterogeneous and may be the result of various causes, either by the virus itself or by other factors such as co-infections, immunosuppression, drugs, pressure injuries related to advanced airway management or due to prone position. The dentist must be aware of the main manifestations that a severe COVID-19 patient may present to achieve a timely diagnosis and treatment.

## Acknowledgments

 The authors wish to thank Dr. Cesar Luna Rivero, Head of the Pathology Service of the National Institute of Respiratory Diseases, for his support in histopathological interpretation.

## Competing Interests

 The authors declare that they have no conflict of interest in this work.

## Ethical Approval

 This study was reviewed and approved by the Ethics Committee of the National Institute of Respiratory Diseases “Ismael Cosío Villegas” and was carried out in accordance with the provisions of the Declaration of Helsinki of 2013

## Funding

 The authors declare that they have not received funding for the preparation of this work.
